# Association of recurrence patterns and outcome with HR and HER2 status in patients with resected brain metastases from breast cancer

**DOI:** 10.1002/ijc.70407

**Published:** 2026-02-25

**Authors:** Jonathan Weller, Sophie Katzendobler, Frederic Thiele, Anna Riesberg, Patrick N. Harter, Frederick Klauschen, Rachel Wuerstlein, Stephan Schoenecker, Montserrat Pazos Escudero, Robert Forbrig, Niklas Thon, Florian Ringel, Michael Weller, Emilie Le Rhun, Veit M. Stoecklein

**Affiliations:** ^1^ Department of Neurosurgery LMU University Hospital, LMU Munich Munich Germany; ^2^ Department of Neurology LMU University Hospital, LMU Munich Munich Germany; ^3^ Center for Neuropathology and Prion Research LMU Munich Munich Germany; ^4^ German Consortium for Translational Cancer Research (DKTK) Partner Site Munich Heidelberg Germany; ^5^ Institute of Pathology LMU Munich Munich Germany; ^6^ Department of Obstetrics and Gynecology, Breast Center and CCC Munich LMU University Hospital, LMU Munich Munich Germany; ^7^ Department of Radiotherapy and Radiation Oncology LMU University Hospital, LMU Munich Munich Germany; ^8^ Department of Neuroradiology LMU Munich Munich Germany; ^9^ Department of Neurosurgery, Knappschaft Kliniken University Hospital of Bochum Bochum Germany; ^10^ Department of Neurology University Hospital and University of Zurich Zurich Switzerland; ^11^ Department of Medical Oncology and Hematology University Hospital and University of Zurich Zurich Switzerland

**Keywords:** hormone receptor, human epidermal growth receptor 2, radiotherapy, triple‐negative, tumor resection

## Abstract

Advanced breast cancer is associated with the development of brain metastases in 20%–40% of patients. Differential post‐radiotherapy recurrence patterns depending on hormone receptor (HR) and human epidermal growth receptor 2 (HER2) status have been reported. We investigated recurrence patterns after microsurgical resection stratified for HR and HER2 expression. The institutional database was screened for patients who had undergone tumor resection for breast cancer brain metastases between 2013 and 2023. Patient and imaging data were analyzed. Response Assessment in Neuro‐Oncology (RANO) guidelines were applied. Sixty‐seven patients were identified. Nineteen patients (28%) were diagnosed with HR+/HER2−, 31 patients (46%) with HER2+, and 17 patients (25%) with triple‐negative (TN) brain metastases. Local, i.e., in or adjacent to the resection cavity, or distant brain‐specific progression‐free survival (PFS) was shortest in patients with TN status, followed by patients with HER2+ and HR+/HER2− brain metastases (median: 170 vs. 419 vs. 1152 days; *p* < .01). Patients with HER2+ brain metastases showed earlier local progression than patients with HR+/HER2− status (HR 0.25; 95% CI 0.08–0.75; *p* = .01). The receptor status of the brain metastases diverged from the primary tumor in 13 patients (21%). In five patients (8%), a newly gained HR or HER2 expression was detected. Post‐surgery recurrence patterns of breast cancer brain metastases are associated with the tumor biology. TN brain metastases show earliest local and distant recurrence, confirming the pressing need for better local and systemic treatments. As HER2+ brain metastases tend to recur locally, refinement of local strategies might be warranted.

## Graphical abstract

What's new?

While treatment strategies for solid brain metastases have been relatively uniform across cancer subtypes, tailored approaches are warranted. In this observational study, intracranial recurrences after microsurgical resection occurred significantly earlier and more frequently at distant sites in patients with triple‐negative breast cancer brain metastases than those with HR+ and HER2+ brain metastases. Patients with HER2+ brain metastases exhibited local recurrence more frequently than patients with HR+ brain metastases. Over a fifth of patients showed discordant receptor status between the brain metastasis and primary breast cancer. The findings may help tailor therapeutic approaches based on intracranial recurrence patterns and biological markers.

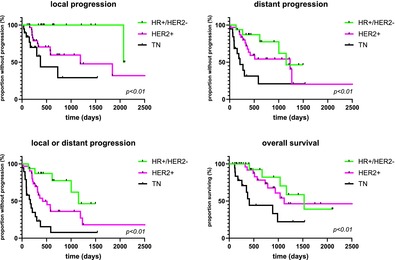

**Abbreviations**
EANO: European Association of Neuro‐Oncology
ER: estrogen
ESMO: European Society for Medical Oncology
FISH: fluorescence in situ hybridization
HER2: human epidermal growth receptor 2
HR: hormone receptor
IHC: immunohistochemistry
KPS: Karnofsky performance status
MRI: magnetic resonance imaging
PFS: progression‐free survival
PR: progesterone receptors
RANO: Response Assessment in Neuro‐Oncology
STX‐VMAT: stereotactic volumetric modulated arc therapy
TN: triple‐negative
WBRT: whole brain radiation therapy

## 1 INTRODUCTION

Brain metastases cause high morbidity and mortality among patients with metastatic breast cancer. Approximately 20%–40% of patients with metastatic breast cancer develop brain metastases.^1^ Despite multimodal treatment, potentially comprising surgery, radiotherapy (RT), chemotherapy, targeted therapy, and immunotherapy, prognosis is poor.^2^, ^3^, ^4^ The decision making for or against neurosurgical metastasectomy depends on various patient‐ and disease‐related factors. Besides patient preference, the clinical status, systemic disease control, and neurological impairment must be considered. Location and mass effect of the brain metastases further shape multidisciplinary tumor board recommendations.^5^, ^6^

The choice of primary therapy and prognosis in patients with breast cancer are largely determined by the expression of hormone receptors (HR) and human epidermal growth factor receptor 2 (HER2).^7^, ^8^, ^9^, ^10^ HR positivity refers to the expression of estrogen (ER) or progesterone receptors (PR), or both, and tumors expressing these receptors might be amenable to endocrine‐based therapy. Medication blocking or degrading HR or therapies lowering systemic estrogen levels with or without ovarian suppression are used.^7^ Another important prognostic and predictive marker is the expression of HER2 that can be found in approximately 20% of patients with breast cancer.^11^ While untreated HER2+ breast cancer behaves aggressively, targeted therapy with monoclonal antibodies, antibody‐drug conjugates or kinase inhibitors may result in long‐term disease control.^7^ Breast cancer lacking HR and HER2 is termed triple‐negative (TN). These tumors, accounting for 10%–15% of breast cancers, are less responsive to systemic treatment.^12^, ^13^, ^14^

RT is a standard treatment for brain metastases, with or without prior surgical intervention.^5^, ^15^ Local recurrence rates after whole brain radiation therapy (WBRT) or stereotactic radiosurgery have been reported to be higher in patients with HER2+ breast cancer while distant recurrence is more frequent in patients with TN breast cancer.^16^

Here we set out to evaluate recurrence patterns of resected brain metastases by applying Response Assessment in Neuro‐Oncology (RANO‐BM) criteria and stratifying according to HR and HER2 expression.^17^ We hypothesized that recurrence patterns differ between biological subtypes and that neurosurgical and other tumor‐specific therapies might need to be tailored accordingly.

## 2 METHODS

### 2.1 Patient selection, therapy and follow‐up

The institutional database of the Ludwig Maximilian University Hospital was screened for patients diagnosed through histological sampling with one or more brain metastases from breast cancer that had been resected between the years 2013 and 2023. Only patients having undergone tumor resection were included. Breast cancer diagnosis had to be established by histological sampling. Histopathological diagnostics were performed at the Center for Neuropathology and the Institute of Pathology of the Ludwig Maximilian University Hospital. HR status was assessed by immunohistochemistry. ER and PR were tested. HR status was termed positive if at least 1% of the tumor nuclei exhibited HR expression, i.e., ER and/or PR, on immunohistochemical staining.^7^, ^18^, ^19^ HER2 status was investigated by immunohistochemistry (IHC) and Fluorescence in situ hybridization (FISH) and respective scores for positivity were assessed: IHC 0–1+: HER2 negative; IHC 2+ with FISH negative: HER2 negative; IHC 2+ with FISH positive: HER2 positive; IHC 3+: HER2 positive.^7^, ^19^, ^20^ Patients with IHC 1+ or IHC 2+ and FISH negative were termed HER2‐low. Brain metastases negative for HER2, ER and PR were termed TN. Patients with positive staining for all receptors were termed triple‐positive. Recommendations for surgical tumor removal were given by a multidisciplinary tumor board. For patients diagnosed between 2017 and 2021, EANO (EANO, European Association of Neuro‐Oncology) Guidelines were followed.^6^ For patients diagnosed between 2021 and 2023, the EANO‐ESMO (ESMO, European Society for Medical Oncology) Clinical Practice Guidelines were applied.^5^ Depending on the clinical status of the patient, surgical removal was considered indicated in case of large tumor volumes, mass effect by the surrounding edema and acute symptoms. Postoperatively, a baseline cranial magnetic resonance imaging (MRI) was performed within 72 h after surgery. RT was initiated in all patients if not contraindicated or declined by the patient. Following RT, routine intervals for clinical and MRI follow‐up were 3 months in case of stable disease. Systemic progression was assessed by whole‐body contrast‐enhanced computer tomography in 3‐month intervals. Leptomeningeal metastases were assessed according to EANO‐ESMO recommendations.^21^

Patient and disease related data were captured (Table 1). Primary tumor control was defined based on clinical or radiological evidence of recurrence at the primary breast cancer site at the time of brain metastasis diagnosis. Patients without signs of local recurrence were classified as having a “controlled” primary tumor, whereas those with documented local recurrence were considered “uncontrolled.” Extracranial systemic tumor control was defined as the absence (“controlled”) or presence (“uncontrolled”) of progressive extracranial systemic tumor manifestations, as determined by clinical documentation and imaging studies at the time of brain metastasis diagnosis. Gross total resection was defined as removal of all contrast‐enhancing tumor tissue as confirmed by postoperative MRI and central review. Brain‐specific progression‐free survival (PFS) was assessed according to RANO‐BM criteria.^17^ Local progression was defined as within or adjacent to the resection cavity. Distant progression was defined as intracranial progression that was not spatially associated with the resection cavity. A threshold of 2 mm minimum distance from the resection cavity was set for distinction between local and distant brain‐specific progression. Overall survival was defined as the day of surgery to the day of death.

**TABLE 1:** Patient characteristics.

Parameter	
Patients, *n* (%)	67 (100)
Age at diagnosis (years)	
Median	54
Range	32–86
KPS at admission	
Median	90
Range	60–100
Number of brain metastases	
Median	1
Range	1–14
Laterality of the brain metastasis	
Left hemisphere	31 (46)
Right hemisphere	34 (51)
Bihemispheric	2 (3)
Location of resected brain metastases	
Frontal	22 (33)
Temporal	5 (8)
Parietal	9 (14)
Occipital	4 (6)
Posterior fossa	24 (36)
Overlapping^a^	3 (5)
Gross total resection	
Yes	55 (82)
No	12 (18)
Postoperative radiotherapy	
Yes	55 (82)
WBRT	18 (33)
Focal radiotherapy	37 (67)
No	12 (18)
Brain progression	
Yes	40 (60)
No	27 (40)
Tumor biology of the brain metastasis	
HR+/HER2−	19 (28)
HER2+	31 (46)
Triple‐negative	17 (25)
Tumor biology of the breast cancer	
HR+/HER2−	18 (27)
HER2+	28 (42)
Triple‐negative	16 (24)
HR‐/HER2+ and HR+/HER2− foci	1 (2)
Missing data	4 (6)

Abbreviations: HER2, human epidermal growth receptor 2; HR, hormone receptor; KPS, Karnofsky performance status; WBRT, whole brain radiotherapy.

^a^ Overlapping refers to multiple affected lobes.

### 2.2 Statistical analyses

Statistical analyses were conducted utilizing the statistical software program GraphPad Prism 10.4.0 (GraphPad Software Inc.). Sankey diagrams were computed by utilizing the browser‐based SankeyMATIC software. The patient cohort was split according to the tumor biology of the brain metastasis into three groups: HR+/HER2−, HER2+, or TN. Patient characteristics of the groups were described by absolute values, percentages, means, medians, and range. To test distributions, D'Agostino‐Pearson normality test was applied. For comparison of parametric data, Students *t*‐test and ANOVA were applied. Categorical data were compared by computing Fisher's exact test and *χ* ^2^‐test. PFS and overall survival analyses were performed by the Kaplan–Meier method. Patient and disease‐related data were subjected to univariate and multivariate Cox proportional hazards model analyses. *p*‐values ≤.05 were considered significant.

## 3 RESULTS

### 3.1 Study population

**FIGURE 1:**
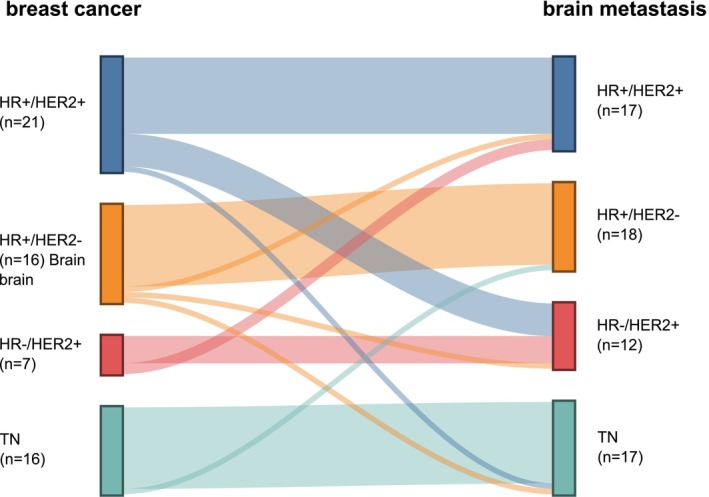
Sankey diagram. The biological features of breast cancers and their respective brain metastases are shown. Of 62 patients with complete biological data, 13 patients (21%) showed a diverging tumor biology in the brain metastasis. Of 23 initially HR‐ breast cancers, newly acquired HR positivity was seen in three patients (13%). Of 34 patients with HER2− breast cancer, two (6%) showed HER2+ brain metastasis. HR, hormone receptor; HER2, human epidermal growth receptor 2; TN, triple‐negative.

The study comprised 67 female patients (Table 1; Figure S1). Median Karnofsky performance status (KPS) at diagnosis of the brain metastases was 90 (range 60–100). The median age was 54 years (range 32–86 years). Mean follow‐up from neurosurgical resection was 27 months (range 3–138). No patient had brain metastases at the initial diagnosis of breast cancer. No patient was diagnosed with leptomeningeal metastases prior to or at the time of neurosurgical tumor resection. The median time from breast cancer diagnosis to brain metastasis was 52 months (range 6–362). In 31 patients (46%), the resected metastasis was located in the left hemisphere and in 34 patients (51%) in the right hemisphere. In two patients (3%), a bihemispheric metastasectomy was performed. In 43 patients (64%), the resected brain metastasis was located supratentorially. The posterior fossa was affected in 24 patients (36%). Forty‐three patients (64%) demonstrated a singular or solitary metastasis at diagnosis. Nine patients (13%) were diagnosed with two brain metastases and 15 patients (22%) had ≥3 brain metastases. Nineteen of 67 patients (28%) had HR+/HER2− brain metastases, 31 patients (46%) were diagnosed with HER2+ brain metastases and 17 patients (25%) showed TN brain metastases (Table 1). A HER2‐low status was seen in 16 patients (24%). ER was detected in 18 of 19 patients (95%) with HR+/HER2− brain metastases, PR expression was found in 10 patients (53%). An ER low positive status, i.e., only 1%–10% of cells stained ER positive, was found in five patients (26%), but only two of these were also HER2−.^18^ One patient with three brain metastases showed diverging immunohistochemical stainings among the brain metastases: one brain metastasis showed ER expression (10%) and two were HR−; one brain metastasis had a HER2+ score of 1+ and two were HER2−. In 62 patients (93%), the tumor biology of the primary breast cancer was known (Table 1). Regarding HR and HER2 status, the breast cancer matched the brain metastasis in 49 patients (79%). In 13 patients (21%), the tumor biology diverged with a newly gained expression of HR or HER2 detected in five patients (8%) and a loss of either receptor expression in eight patients (13%) (Figure 1).

### 3.2 Tumor‐specific treatment

All patients had undergone resection of at least one brain metastasis. In 61 patients (91%), one brain metastasis was resected. In four patients (6%), two spatially separate metastases were resected in one surgery. In two patients (3%), three brain metastases were resected at once. Gross total resection of contrast‐enhancing tumor assessed by MRI was achieved in 55 patients (82%). In 12 patients (18%), contrast‐enhancing foci suspicious for residual tumor were seen on postoperative MRI. Residual tumor was seen in six of 19 patients (32%) with HR+ brain metastases, four of 31 patients (13%) with HER2+ brain metastases, and two of 17 patients (12%) with TN brain metastases (*p* = .21). The median number of systemic therapies received prior to the brain metastasis was 3 (range 0–14) (Table S1). Twenty‐eight patients (42%) were on systemic treatment when the brain metastasis was diagnosed. In 62 patients, data on systemic therapy after diagnosis of the brain metastasis was available. Forty‐seven patients (76%) received systemic treatment after diagnosis of the brain metastasis. Fifteen patients (24%) did not receive further systemic therapy. Six patients had already received RT once for the treatment of brain metastases before surgery, and four of these patients did not receive additional RT after tumor resection. Postoperative RT was administered in 55 patients (82%). Fractionated stereotactic volumetric modulated arc therapy‐based (STX‐VMAT) tumor bed RT was applied in 37 patients. WBRT was applied to 18 patients, with 5 patients additionally receiving tumor bed boosts. The median applied RT dose was 28 Gy (range 20–56) applied in daily fractions of 5 Gy. In patients with HER2‐low status and after diagnosis of the brain metastasis, systemic treatment was given in 11 patients (69%). At that time, no patient was treated with trastuzumab deruxtecan.

### 3.3 Patterns of intracranial recurrence, extracranial tumor status and Kaplan Meier estimates

**FIGURE 2:**
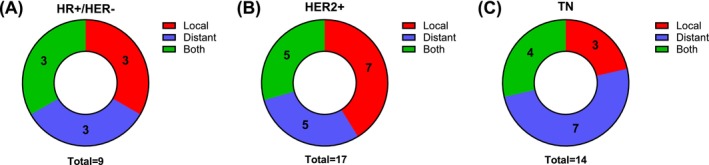
Local versus distant brain‐specific progression. Progression was determined on contrast‐enhanced MRI. A tumor recurrence in the resection cavity or within 2 mm of the surrounding brain parenchyma was termed “local.” Tumor recurrences without spatial relation to the resection cavity were termed “distant.” The rate of local progression was lower in HR+/HER2− metastases (A) than in HER2+ metastases (B) and similar to TN tumors (C). MRI, magnetic resonance imaging; HR+/HER2−, hormone receptor positive and human epidermal growth receptor 2 negative; HER2+, human epidermal growth receptor 2 positive; TN, triple‐negative.

Intracranial recurrences were observed in 40 patients (60%). Combined local and distant progression within the brain was observed in 12 patients (18%). A local recurrence without distant progression was seen in 13 patients (19%). An exclusively distant progression without local recurrence was seen in 15 patients (22%) (Figure 2). Median brain‐specific PFS was 496 days. The primary tumor status was controlled in 56 patients (84%) at the time of brain metastasis diagnosis. Systemic, extracranial metastases had already been diagnosed in 26 patients (39%) prior to the development of brain metastases and in 16 patients (24%), the systemic, extracranial status was uncontrolled at the time of brain metastasis diagnosis. Median overall survival was 1527 days. At database closure, 22 deaths (33%) had been reported. Non‐neurological death was suspected in nine patients (13%) while neurological death was suspected in three patients (5%). In 10 patients (15%), there was no sufficient data on the ultimate cause of death.

**FIGURE 3:**
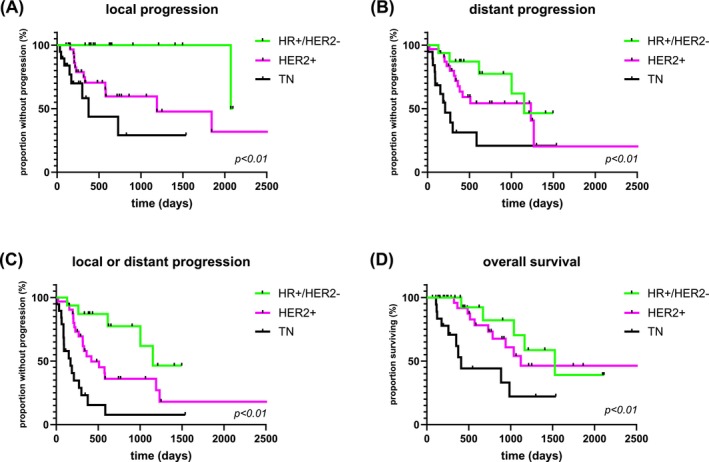
Kaplan–Meier curves for progression‐free and overall survival. Time to local progression (A), distant progression (B), any brain progression (C), and death (D) stratified by tumor biology (HR+/HER2−, HER2+ and TN). HR+/HER2−, hormone receptor positive and human epidermal growth receptor 2 negative; HER2+, human epidermal growth receptor 2 positive; TN, triple‐negative.

In patients with TN brain metastases, median PFS was shortest, followed by patients with HER2+ and HR+/HER2− brain metastases (median, in days: 170 vs. 419 vs. 1152; *p* < .01). TN status was associated with the shortest absence from distant progression (median, in days: 210 vs. 1233 vs. 1152; *p* < .01). Comparing patients with HR+/HER2− and HER2+ brain metastases, there was no difference in absence from distant progression (*p* = .24), but patients with HER2+ brain metastases showed shorter absence from local progression with 1191 versus 2084 days (HR 0.25; 95% CI 0.08–0.75; *p* = .01) (Figure 3). In the subgroup of patients with HER2 low brain metastases (*n* = 16; 24%), median PFS was 582 days. Absence from local progression was 375 days in patients with TN brain metastases. There was no difference in PFS between patients with HR+/HER2+ and HR−/HER2+ brain metastases (*p* = .51). Two patients (3%) with ER expression of 1%–9% and HER2− status, i.e., ER low tumors, were identified. These patients showed a PFS of 145 and 1115 days compared to the median PFS of 170 days in TN patients. Patients with biological changes regarding HR or HER2 in their brain metastases did not show shorter PFS (*p* = .36). Comparing WBRT to stereotactic RT, there was no significant difference in brain‐specific PFS (median, in months: 20 vs. 19; HR 0.87; 95% CI 0.41–1.82, *p* = .69). There was no difference in local control (HR 0.74; 95% CI 0.29–1.89; *p* = .51) or distant control (HR 0.76; 95% CI 0.22–2.6; *p* = .66). Exclusively local progression occurred in six of 37 patients (16%) having received STX‐VMAT and in three of 18 patients (17%) treated with WBRT (*p* > .99). Respective rates for exclusively distant progression were nine of 37 patients (24%) in the STX‐VMAT group versus five of 18 patients (28%) in the WBRT group (*p* > .99). A concurrent local and distant progression was seen in six of 37 patients (16%) with stereotactic radiosurgery and in four of 18 patients (22%) with WBRT (*p* = .71).

Fourteen patients (21%) developed leptomeningeal metastases in the disease course after surgery. Cerebrospinal fluid confirmed the diagnosis in eight patients (12%) (EANO‐ESMO Type I) whereas clinical signs and MRI studies were suggestive of leptomeningeal metastases in six patients (9%) (EANO‐ESMO Type II).^21^ Patients with HR+/HER2−, HER2+ and TN brain metastases developed leptomeningeal metastases at rates of 2/19 (11%), 6/31 (19%), and 6/17 (35%) (*p* = .21). There was no significant difference in the number of patients developing leptomeningeal metastases between the two RT groups (WBRT 5/18 [28%]; stereotactic radiosurgery 7/37 [19%]; *p* = .5).

### 3.4 Uni‐ and multivariate analyses of PFS

Factors tested for association with PFS and OS were age, KPS, primary tumor control and extracranial tumor control at diagnosis of the brain metastasis, number of brain metastases, gross total versus subtotal resection, biological status (HR and HER2), systemic treatment, and application of postoperative RT. In univariate analysis, age, KPS, tumor biology, and RT showed an association with PFS (Table 2). In multivariate analysis, a TN status (HR 0.47; 95% CI 0.27–0.82; *p* < .01) and no administration of RT (HR 0.38; 95% CI 0.16–1.0; *p* = .04) were associated with shorter PFS (Table 2).

**TABLE 2:** Univariate and multivariate analyses of factors potentially associated with brain‐specific progression‐free survival.

Factor	Brain‐specific progression		
	HR	95% CI	*p*‐value
Univariate analyses			
Age^a^	0.97	0.94–1.0	.04^b^
KPS^a^	1.04	1.0–1.07	.04^b^
Number of brain metastases^a^	0.91	0.7–1.09	.42
Gross total versus partial resection	0.61	0.29–1.44	.22
Primary tumor control (uncontrolled vs. controlled)	0.56	0.26–1.41	.2
Systemic, extracranial tumor control (uncontrolled vs. controlled)	0.54	0.18–1.23	.17
Systemic treatment after diagnosis of the brain metastasis	0.8	0.4–1.75	.56
Tumor biology (HR+ vs. HER+ vs. TN)^a^	0.48	0.3–0.75	<.01^b^
Postoperative RT versus no RT	0.31	0.13–0.8	<.01^b^
Multivariate analyses			
Age^a^	1.0	0.97–1.03	.83
KPS^a^	1.03	0.99–1.07	.17
Tumor biology (HR+ vs. HER+ vs. TN)^a^	0.47	0.27–0.82	<.01^b^
Postoperative RT versus no RT	0.38	0.16–1.0	.04^b^

Abbreviations: CI, confidence interval; HER2, human epidermal growth receptor 2; HR, hazard ratio; HR+, hormone receptor positive; KPS, Karnofsky performance status; RT, radiotherapy; TN, triple‐negative.

^a^ Continuously scaled.

^b^ Statistically significant.

### 3.5 Univariate analyses of overall survival

In univariate analyses, primary tumor control and systemic, extracranial tumor control showed an association with overall survival (HR 0.3; 95% CI 0.13–0.79; *p* = .02 and HR 0.4; 95% CI 0.17–0.97; *p* = .04). There was a borderline association between tumor biology of the brain metastases and overall survival (HR 0.55; 95% CI 0.3–0.99; *p* = .05) (Table 3). In multivariate analysis, only receptor status was associated with overall survival (HR 0.52; 95% CI 0.28–0.93; *p* = .03).

**TABLE 3:** Univariate and multivariate analyses of factors potentially associated with overall survival.

Factor	Overall survival		
	HR	95% CI	*p*‐value
Univariate analyses			
Age^a^	0.97	0.93–1.01	.09
KPS^a^	0.98	0.94–1.02	.33
Number of brain metastases^a^	1.01	0.84–1.3	.41
Gross total versus partial resection	0.48	0.2–1.26	.11
Primary tumor control (uncontrolled vs. controlled)	0.3	0.13–0.79	.02^b^
Extracranial tumor control (uncontrolled vs. controlled)	0.4	0.17–0.97	.04^b^
Systemic treatment after diagnosis of the brain metastasis	0.87	0.34–2.68	.79
Tumor biology (HR+ vs. HER+ vs. TN)^a^	0.55	0.3–0.99	.05^b^
Postoperative RT versus no RT	0.74	0.25–3.19	.64
Multivariate analyses			
Primary tumor control (uncontrolled vs. controlled)	0.38	0.15–1.06	.06
Systemic, extracranial tumor control (uncontrolled vs. controlled)	0.41	0.16–1.06	.06
Tumor biology (HR+ vs. HER+ vs. TN)^a^	0.52	0.28–0.93	.03^b^

Abbreviations: CI, confidence interval; HER2, human epidermal growth receptor 2; HR, hazard ratio; HR+, hormone receptor positive; KPS, Karnofsky performance status; RT, radiotherapy; TN, triple‐negative.

^a^ Continuously scaled.

^b^ Statically significant.

## 4 DISCUSSION

While treatment strategies for patients with solid brain metastases across different cancer subtypes have historically been relatively uniform, more tailored approaches are warranted in the future. These tailored approaches might be influenced by the pattern and timing of intracranial recurrences. As different post‐RT recurrence patterns (without prior surgical resection) of breast cancer brain metastases depending on the tumor biology have been described, the present observational study aimed to explore whether these findings also apply to a surgically treated cohort.^16^ We report a strong association between time to recurrence and its pattern with the tumor biology of the resected brain metastases. Patients diagnosed with TN brain metastases showed earlier recurrences than patients with HR+/HER2− or HER2+ brain metastases (Figure 3). Patients with HER2+ tumors showed earlier local progression than patients with HR+/HER2− brain metastases, even with consideration of post‐operative RT. Although systemic treatment can aid in controlling the cancer and improving overall survival in patients with HER2+ metastatic breast cancer, even with brain metastases, these patients might require refined local treatment strategies.^22^, ^23^ Studies on supramarginal resection of brain metastases are scarce.^24^, ^25^, ^26^ Current surgical strategies mostly aim at resecting contrast‐enhancing foci on MRI. Some studies suggest infiltration of brain parenchyma beyond contrast‐enhancement in patients with brain metastases.^27^, ^28^, ^29^ Especially in patients with brain metastases in non‐eloquent regions from HER2+ breast cancer, supramarginal tumor resection might be indicated to prevent early local recurrence. This suggestion remains hypothesis‐generating and warrants further prospective investigation. Further studies on tumor margins and infiltration zones beyond contrast‐enhancing foci on MRI are warranted to address this question.

Development of leptomeningeal metastases is associated with poor survival.^30^ In the further disease course after the neurosurgical resection of the brain metastasis, 14 patients (21%) developed leptomeningeal metastases. Among patients with newly diagnosed intracranial metastases from breast cancer, approximately 11% have been reported to show concurrent leptomeningeal metastases.^31^ In our study, patients with concurrent leptomeningeal metastases at first diagnosis of intracranial manifestation did not undergo tumor resection and are thus not represented here. Irrespective of the patterns of care, patients with brain metastases are more likely also to develop leptomeningeal disease later during the clinical course.

Genomic alterations with potential clinical implications have been reported to differ in 53% between primary cancer and brain metastasis.^32^ Here, one in five patients showed a diverging tumor biology of the primary cancer compared to the brain metastasis. The receptor status of brain metastases might have major implications for treatment decision making. Endocrine therapy in patients with HR+ breast cancer and brain metastases has been associated with prolonged survival.^33^ Systemic treatment with trastuzumab deruxtecan might be associated with prolonged survival in patients with brain metastases from HER2+ breast cancer, potentially even in patients with HER2‐low status.^34^, ^35^, ^36^ In our cohort, patients with HER2‐low status showed an intermediate PFS, similar to HER2+ patients. Because all patients were diagnosed between the years 2013 and 2023, none had received trastuzumab deruxtecan at the diagnosis of brain metastasis. Stereotactic biopsy techniques allow for low‐risk tissue acquisition in patients with brain tumors and might be indicated in these patients.^37^, ^38^ Evaluation of improved surgical strategies according to genomic features would require knowledge of tumor biology. Implementation and refinement of preoperative diagnostics such as artificial intelligence‐based MRI analysis and liquid biopsies might provide biological information on brain metastases prior to surgery.^39^, ^40^, ^41^ Improvement of time‐efficient intraoperative diagnostics such as stimulated Raman histology and nanopore sequencing might further facilitate intraoperative diagnosis and adjustment of surgical strategies.^42^, ^43^ A major limitation of this study is its retrospective nature. By only including patients having undergone tumor resection, a selection bias toward a cohort of patients with surgically accessible brain metastases and a better clinical status might be introduced. This reduces the external validity of the findings. The study is further limited by the sample size, especially when stratifying for receptor status. Conclusions on the differential role of stereotactic radiosurgery versus WBRT can also not be derived due to limited sample size. The present study is observational in nature and should be interpreted as hypothesis‐generating. While our findings support previously described subtype‐specific recurrence patterns, they emphasize the need for integrative molecular and imaging‐based approaches in future studies to understand the mechanisms driving local and distant intracranial progression. As our cohort largely reflects patients treated between 2013 and 2023, before the widespread adoption of trastuzumab deruxtecan and other novel HER2‐targeted agents, our findings represent recurrence dynamics under earlier treatment paradigms. Future studies should assess whether these associations remain valid in patients receiving contemporary HER2‐directed therapies. Determining cause of death can be difficult in patients with metastatic breast cancer. In systemically progressive disease, the cause of death is often uncertain, as patients gradually deteriorate and transition to palliative care. We therefore focused primarily on brain‐specific PFS as a more direct measure of intracranial disease dynamics. However, it should be acknowledged that the relationship between progression‐free and overall survival in this setting remains uncertain, which may limit the broader prognostic interpretation of our findings.

In summary, we report earlier and oftentimes distant and disseminated intracranial recurrence in patients with TN brain metastases. In these patients and if multiple brain metastases are present, evaluation of new RT or radionuclear approaches is warranted. HER2+ brain metastases showed a higher likelihood of local recurrence compared to HR+/HER2− metastases, which may justify further exploration of surgical margin strategies in this subgroup. Finally, our findings reinforce the relevance of reassessing receptor status in brain metastases, as discordance from the primary tumor may influence future treatment planning.

## AUTHOR CONTRIBUTIONS

**Jonathan Weller:** Conceptualization; investigation; writing – original draft; visualization; formal analysis; data curation; software. **Sophie Katzendobler:** Data curation; resources. **Frederic Thiele:** Formal analysis; data curation. **Anna Riesberg:** Data curation; resources. **Patrick N. Harter:** Investigation; writing – review and editing; methodology. **Frederick Klauschen:** Investigation. **Rachel Wuerstlein:** Conceptualization; writing – review and editing; validation; methodology; formal analysis; supervision. **Stephan Schoenecker:** Investigation; methodology; validation. **Montserrat Pazos Escudero:** Conceptualization; methodology; writing – review and editing. **Robert Forbrig:** Investigation; data curation. **Niklas Thon:** Conceptualization; writing – review and editing. **Florian Ringel:** Conceptualization; investigation; writing – review and editing; supervision. **Michael Weller:** Conceptualization; investigation; methodology; validation; writing – review and editing; writing – original draft; supervision; project administration. **Emilie Le Rhun:** Conceptualization; writing – review and editing; project administration. **Veit M. Stoecklein:** Conceptualization; project administration; supervision; data curation; investigation.

## CONFLICT OF INTEREST STATEMENT

Professor Frederick Klauschen is co‐founder of Aignostics, an AI‐based company spin‐off from Charité Universitätsmedizin Berlin. Professor Rachel Wuerstlein has received honoraria and/or travel support and has served as an advisor, consultant, or speaker for Agendia, Amgen, ApogeeVA, Aristo, AstraZeneca, Bayer, Celgene, Clinsol, Daiichi Sankyo, Eisai, Esteve, Exact Sciences, Gilead, Hexal/Sandoz, Jenapharm, Lilly, MSD, Mundipharma, Mylan, Nanostring, Novartis, Onkowissen, Paxman, Palleos, Pfizer, Pierre Fabre, PINK, Riemser, Roche, Seagen, Sidekick, Stemline, Tesaro Bio, Teva, Veracyte, Viatris, Wiley, and Thieme. In addition, RW has collaborated with Digimed, Eickeler, FOMF, Art Tempi, Aurikamed, Clinsol, Pomme Med, iMED Institutmedconcept, MCI, MediSeminar, Medicultus, H + O Communications, New Oncology Concepts, SPCC, streamed up, medupdate GmbH, Lukon, and NGA GmbH. Institutional affiliations of RW include SPCC, WSG, AGO, and ESMO. Professor Michael Weller has received honoraria for advisory board participation and/or consulting from Bayer, CureVac, Hemerion, Iqvia, Medac, Novartis, Novocure, Orbus, Pfizer, Philogen, Roche, and Servier. In addition, institutional research support has been received from Novartis, Quercis, and Versameb. E. le Rhun reports receiving research grants from BMS and Servier. She has received honoraria for lectures, participation in advisory boards, and consulting from AstraZeneca, Daiichi Sankyo, Bayer, Biodexa/Sitoxi, Janssen, Leo Pharma, Medac, myTomorrows, Pfizer, Pierre Fabre, Roche, Seattle Genetics, and Servier. All other authors declare that they have no known competing financial interests or personal relationships that could have appeared to influence the work reported in this paper.

## ETHICS STATEMENT

The study has been approved by the institutional ethics committee of the Ludwig Maximilian University Munich (project number 24‐1009).

## Supporting information


**Data S1.** Supporting Information.

## Data Availability

The data that support the findings of this study are available from the corresponding author upon reasonable request and after approval by the institutional ethics committee of the Ludwig Maximilian University Munich.
