# Supplementary material for: Association of recurrence patterns and outcome with HR and HER2 status in patients with resected brain metastases from breast cancer
Source: Int J Cancer. 2026 Feb 25;159(2):440–9. doi: 10.1002/ijc.70407 (PMC13193514; doi:10.1002/ijc.70407)
Supplement: Supplementary file 1 — Data S1. Supporting Information. [file IJC-159-440-s001.pdf]

Association of recurrence patterns and outcome with HR and HER2 status in patients with resected brain metastases from breast cancer

Jonathan Weller, Sophie Katzendobler, Frederic Thiele, Anna Riesberg, Patrick N. Harter, Frederick Klauschen, Rachel Wuerstlein, Stephan Schoenecker, Montserrat Pazos Escudero, Robert Forbrig, Niklas Thon, Florian Ringel, Michael Weller, Emilie Le Rhun, Veit M. Stoecklein

Table of Contents

Supplementary Table 1: Systemic, tumor-specific therapies administered prior to the development of brain metastases

Supplementary Fig. 1. Patient cohort stratified according to the biological features of the brain metastasis.

Supplementary material

Suppl. Table 1: Systemic, tumor-specific therapies administered prior to the development of brain metastases.*

**Receptor status of the brain metastasis**	**Patients, n (%)**	**Therapy classes**	**Most common agents used prior to the development of brain metastases (number of patients)**
HR+/HER2−	19 (28)	Endocrine therapy ± chemotherapy ± targeted agents (CDK4/6, mTOR inhibitors)	Tamoxifen (6), Letrozole (5), Exemestane (5), Epirubicin/Cyclophosphamide or FEC regimen (5), Paclitaxel (5), Docetaxel (4), Fulvestrant (3), Palbociclib or Ribociclib (3), Everolimus (2)
HER2+	31 (46)	HER2-targeted therapy ± chemotherapy ± endocrine therapy	Trastuzumab (29), Pertuzumab (15), Docetaxel (10), Tamoxifen (9), Paclitaxel (9), Letrozol (5), Goserelin (4), Epirubicin (4), EC (3), Anastrozol (3), Trastuzumab-Emtansine (3), TAC (2)
Triple-negative	17 (25)	Chemotherapy ± immune checkpoint inhibitors	Epirubicin/Cyclophosphamide or FEC regimen (6), Carboplatin (6), Docetaxel (5), Paclitaxel (5), Capecitabine (5), Gemcitabine (2), Atezolizumab (2), Pembrolizumab (1)

*Many patients had more than one prior line of treatment. Bone-targeted agents such as bisphosphonates and denosumab are not included. *HR, hormone receptor; HER2, human epidermal growth factor receptor 2; CDK, cyclin-dependent kinase; mTOR, mechanistic target of rapamycin; FEC, fluorouracil/epirubicin/cyclophosphamide.*

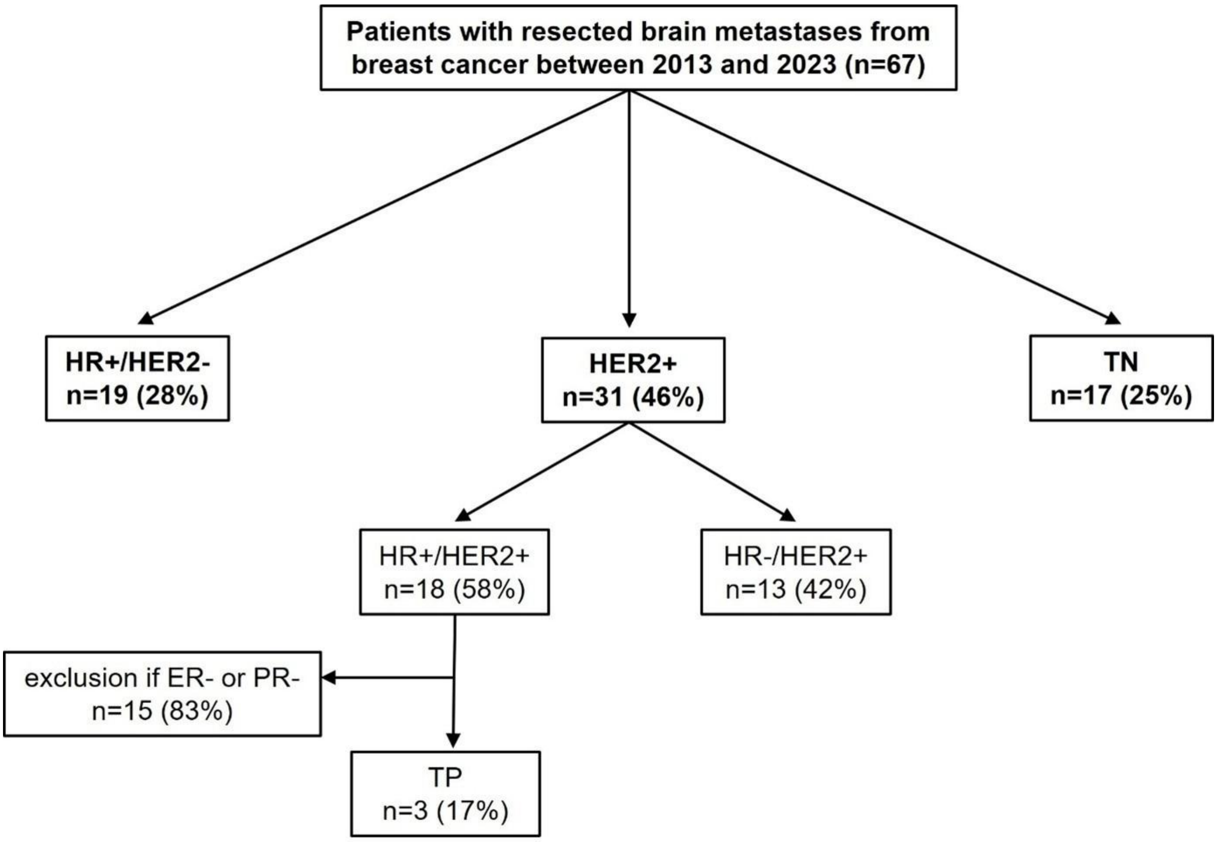

Suppl. Fig. 1. Patient cohort stratified according to the biological features of the brain metastasis. *HR, hormone receptor; HER2, human epidermal growth receptor 2; TN, triple-negative; ER, estrogen receptor; PR, progesterone receptor; TP, triple-positive.*
